# Ankle dorsi- and plantar-flexion torques measured by dynamometry in healthy subjects from 5 to 80 years

**DOI:** 10.1186/1471-2474-14-104

**Published:** 2013-03-22

**Authors:** Amélie Moraux, Aurélie Canal, Gwenn Ollivier, Isabelle Ledoux, Valérie Doppler, Christine Payan, Jean-Yves Hogrel

**Affiliations:** 1 Institut de Myologie, UPMC UM 76, INSERM U 974, CNRS UMR 7215, 75651, Paris Cedex 13, France

**Keywords:** Muscle strength, Normative data, Prediction model, Outcome measures

## Abstract

**Background:**

Ankle strength is often impaired in some of the most common neuromuscular disorders. Consequently, strength generated around this joint is important to assess, because it has a great impact on balance and gait. The objectives of this study were to establish normative data and predictive equations for both ankle dorsi- and plantar-flexion strength from a population of healthy subjects (children and adults), to assess the reliability of the measurements and to study the feasibility of using a novel dynamometer on a group of patients with a neuromuscular disorder.

**Methods:**

Measurements of maximal isometric torque for dorsi- and plantar-flexion were performed on 345 healthy subjects from 5 to 80 years of age. The feasibility of the method was tested on nine patients diagnosed with type 2A limb girdle muscular dystrophy.

**Results:**

The results documented normal strength values depending on gender and age on ankle dorsi- and plantar-flexion. The reliability of the technique was good with no evaluator effect and a small learning effect. The dynamometer was found suitable in the group of patients, even very weak.

**Conclusions:**

The device developed was both reliable and accurate in assessing both ankle dorsi-flexion and plantar-flexion torque measurements from weak patients and children to strong healthy adults. Norms and predictive equations are provided for these two muscle functions.

## Background

Neuromuscular disorders are associated with a progressive loss of muscle strength. Knowing the defect of strength that the patient suffers from is essential to assess the evolution of the disease and may be helpful in quantifying the beneficial effects of different therapeutic strategies. As new therapies emerge, finding reliable and sensitive outcome measures has become a priority in the neuromuscular community. Ankle strength is often impaired in many of the most common neuromuscular disorders and is an important function to monitor as it has a great impact on both balance or gait [1,2]. For instance, in Charcot Marie Tooth (CMT) disease, ankle dorsi-flexion weakness has been demonstrated to influence motor function [3]. In Duchenne muscular dystrophy (DMD), Bakker and al. found ankle dorsi-flexion to be a good predictor for the loss of ambulation [4]. Dorsi-flexion is also affected in several adult neuromuscular disorders such as myotonic dystrophy type 1 [5] or inclusion body myositis [6].

Ankle strength measurement has traditionally been performed using manual muscle testing (MMT), hand held dynamometry or isokinetic torque measurement systems. MMT is easy to perform as it does not require any specific equipment but it has a low reliability [7] and low sensitivity [8,9]. Hand held dynamometry has the advantage over MMT of being more sensitive but the reproducibility and accuracy of measurements is dependent upon standardization of joint position which can be problematic for neuromuscular disorders such as DMD or spinal muscular atrophy (SMA) where patients present contractures [7,10]. Another limitation of hand held dynamometry is that the tester has to be stronger than the patient to hold the dynamometer stationary [11]. This can be particularly problematic for the evaluation of plantar flexors in adults and in adolescents for whom torque can easily reach values that the evaluator can have difficulties counteracting [12]. By using isokinetic/isometric measurement systems it is possible to accurately standardize joint position [13,14] and to assess strong functions more reliably than MMT or handheld dynamometry. They are easy to use but they have the disadvantage of being rather expensive. Assessing ankle strength in children can also be difficult since the fixation pads are designed for adults and may not stabilize children’s joints accurately [15]. All of these methods have advantages and disadvantages and none have been currently unanimously recognized as a reference measurement method for ankle strength.

Due to the limitations of existing strength measurement systems, several studies have used home-made dynamometers based on strain gauges and designed specifically to measure ankle dorsi-flexion [16-20], ankle plantar-flexion [15,21-23] or both [24-27]. Todd et al. [25] have recently designed a unique dynamometer which can measure both ankle dorsi-flexion and plantar-flexion functions. Solari et al. [26] designed a fixation device to maintain a hand held dynamometer stationary to evaluate ankle strength in patients with CMT disease. Both devices have the advantage of offering easy stabilization of the joint for the measurement of plantar flexors. However the maximal range of measurement of these devices (1 kN and 500 N, respectively) might be too low and limits their applications as plantar-flexion torques can reach values as high as 220 N⋅m [13].

The dynamometer we have designed in the present study was developed to measure both dorsi-flexion and plantar-flexion strengths within a wide range of values, to provide good stabilization of the joint position and to be applicable to subjects of different statures. It can be used in patients with decreased muscle performance as well as healthy children and adults of all ages.

Normative models are useful for the assessment of patients because deficits are generally bilateral and progressive. Patients are expected to change as they age or grow, so tracking clinical changes are affected by these factors. It is therefore useful to express strength as a deficit from normal expected values.

The objectives of this study were to establish normative data and predictive equations for both ankle dorsi- and plantar-flexion strength from a population of healthy subjects, to assess the reliability of the measurements and to study the feasibility of using this dynamometer on a group of patients with a neuromuscular disorder.

## Methods

### Participants

Healthy subjects, male or female, aged between 5 to 80 years old were recruited by advertisements in newspapers, websites, and posters. Exclusion criteria were any history of injury, any disease involving the lower limbs in the last two years, pain or discomfort that affect the lower limbs or the practice of a sport at a national level. Subjects were informed about the terms of the experimental protocol and procedures before giving their written consent. The protocol (namely MyoTools) was approved by the Local Ethics Committee (CPP-Ile de France VI). The dominant side was reported according to the dominant hand defined as the hand with which the subject writes. The feasibility of measurements on patients who demonstrated muscle weakness was assessed by measuring ankle dorsi-flexion and plantar-flexion strength on a group of patients with limb girdle muscular dystrophy type 2A (LGMD2A or calpainopathy) using the same experimental procedure as for healthy subjects. These measures were included into a natural history protocol, also approved by the Local Ethics Committee. All subjects and patients gave informed written consent to participate to the measurement sessions.

### Anthropometric measurements

The height and weight of the subjects were recorded as well as an estimation of the percentage of body fat mass using an impedance metric scale (TBF-543, Tanita Corporation, Arlington Heights, Illinois, USA). The lever arm of both feet was measured as the distance from the head of the fifth metatarsal bone to the lower extremity of the external malleolus. As the centre of the lateral malleolus is usually considered as the centre of rotation of the ankle joint [28], the lower extremity of the external malleolus was assumed to be on the line of projection of the centre of rotation of the ankle joint to the ground.

### Dynamometer description

**Figure 1:**
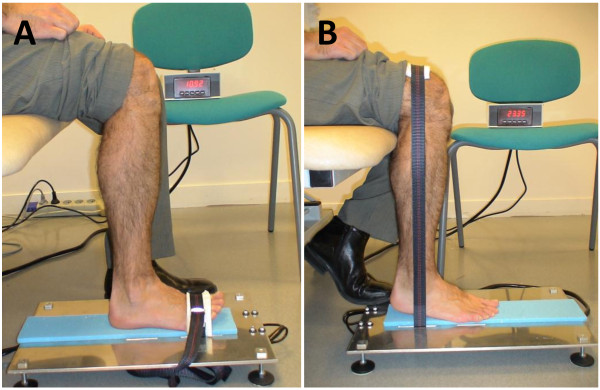
Dorsiflexion (A) and plantarflexion (B) strength measurement.

The ankle dynamometer was home-made and designed to measure the isometric torque generated around the ankle joint in both extension and flexion directions (Figure 1). It consists of an aluminium plate below which are held two load cells (model AG100; Scaime company, France) on which strain is applied through a non-elastic strap going through slots in the plate. The strap is placed and held either on top of the foot at the level of the first metatarsal for dorsi-flexion measurement, or placed distally on the thigh and passed directly over the external malleolus for plantar-flexion measurement (Figure 1). To ensure a safer and stiffer support on the knee, the strap was reinforced with a composite material made of two layers of foam between which was held a stiff but flexible metal sheet. The nominal force measured by the device (combination of two load cells) is 200 decaNewtons (daN); its precision is 0.1 daN and its resolution is 0.01 daN. The dynamometer is easily transportable since its weight is less than 3 kg for small external dimensions (50 × 30 × 10 cm). The transducer signal is conditioned by an electronic board with an onboard analogic low pass filter (cut-off frequency: 10 Hz). A digital screen can either continuously display the force, or retain the maximal value in the extension and flexion directions. A BNC output connector is also provided to record the analog signal of force for feedback or advanced analysis methods of strength. The load cells and their electronic attachments were calibrated in a factory according to strict operating procedures attached to the quality assurance ISO 17025. A certificate of calibration was delivered by the factory. Verification of calibration was performed periodically to ensure accuracy of measurements.

### Experimental procedure

The subjects were seated on a plinth adjustable to the height of the subject in order to obtain a right angle at the hip, knee and ankle joints; the leg being vertical. The foot was flat on the dynamometer. For ankle dorsi-flexion measurement, the top of the foot was tightly strapped to lock the ankle in place. The subject was asked to pull up against the strap to flex the ankle. For ankle plantar-flexion, the strap was placed distally on the thigh and passed directly over the external malleolus. The subject was asked to pull against the strap by extending his ankle while pushing with the sole of their foot and trying to lift the heel. Subjects were verbally encouraged to produce their maximal ankle dorsi-flexion or plantar-flexion strength. Two trials were recorded, consisting of two 2-4 second maximal contractions separated by a 30 second rest period. If the relative difference between these two maximal voluntary contractions (MVC) was within 10%, no additional trial was required. If not, additional trials were proposed until two reproducible MVCs were obtained. The maximal value of the two reproducible trials was retained for analyses. The maximal torque was computed as the product between the linear force and the lever arm. The contra-lateral side was then tested using the same procedure. Dorsi-flexion which is the weakest function was tested first, to enable the subject to familiarize himself with maximum voluntary contraction, and then the plantar-flexion was tested. The side selected for the first measure (i. e. right or left) for each function was randomly selected.

A subgroup of subjects agreed to perform a retest-session for ankle strength. The experimental conditions were the same as for the first session. The evaluator was either the same or another evaluator trained to the experimental procedures. Three independent evaluators performed the measurements to assess reliability. The retest session was performed at least one hour after the first session or planned within 30 days following the test session.

The feasibility of measurements on weak patients was assessed by measuring ankle dorsi-flexion and plantar-flexion on a group of patients with LGMD2A. The experimental procedure was the same as for healthy subjects. Complementary clinical data were also available for the patients such as the Vignos score.

### Statistical analyses

Norms were established in N⋅m by groups of five years of age for younger subjects up to 20 years old and then by groups of 10 years of age. In order to decide whether norms should be established according to the side tested or to the dominant side, the ankle torque values between the right and left sides were compared taking into account the dominance effect. The ankle torque values between the dominant and non-dominant sides in both right-handers and left-handers were compared by means of a paired Student t-test.

To establish predictive equations, children and adults were considered separately. Subjects in the children group were younger than eighteen years old. Stepwise linear regressions were performed to detect the best predicting variables for ankle dorsi-flexion and plantar-flexion maximal strength. Independent variables tested were height, weight, age, sex, body mass index, percentage of body fat. Predictive equations for each function were established separately on right and left sides. The statistical analyses were performed using SPSS (v19.0). The limit of significance for all tests was set at p<0.05.

The difference between test and retest sessions was evaluated by taking into account the evaluator effect and the side effect for each function using a repeated measurements analysis of variance. The standard error of measurement, the coefficient of variation (CVar) and the limits of agreement according to Bland and Altman [29] were calculated. To assess reliability, the intra-class correlation coefficient (ICC) was computed as a single measure ICC with a two-way random-effect model (absolute agreement). The ability of the device to discriminate between two measurements was computed as the smallest detectable difference (SDD) according to Beckerman et al. [30].

Predictive equations were applied to the patients’ data to compute predicted strength values and z-scores for each function and each side.

## Results

### Subjects

Three hundred and forty five subjects were included for the measurement of ankle strength: 57 children under 18 years (28 boys, 29 girls), 288 adults (119 men, 169 women). Their characteristics are given in Table 1. All subjects had dorsi-flexion measurements and 325 had plantar-flexion measurements (a technical issue prevented the performance of plantar-flexion measurements on 20 subjects). Ninety per cent of the subjects were right handed.

**Table 1:** Subjects’ characteristics given as mean (SD)

**Age (years)**	**Sex**	**n**	**Height (cm)**	**Weight (kg)**	**Percentage of body fat mass (%)**	**Left ankle dorsi-flexion (N⋅m)**	**Right ankle dorsi-flexion (N⋅m)**	**Left ankle plantar-flexion (N⋅m)**	**Right ankle plantar-flexion (N⋅m)**
5-9	F	12	127.8 (10.4)	28.3 (7.8)	24.0 (4. 5)	8.2 (2.9)	8.8 (3.0)	39.0 (5.6)	40.2 (6.4)
	M	15	118.0 (8.3)	22.8 (4.6)	14.1 (4.7)	5.6 (2.0)	6.3 (2.3)	33.3 (8.9)	36.4 (12.1)
10-14	F	9	154.1 (11.2)	44.1 (11.5)	23.6 (3.9)	16.6 (3.8)	16.8 (4.6)	79.2 (31.9)	83.4 (30.6)
	M	11	155.5 (10.4)	44.8 (9.9)	16.7 (4.5)	15.8 (6.2)	19.0 (6.0)	81.3 (28.3)	89.4 (31.5)
15-19	F	15	164.6 (5.0)	59.4 (11.4)	26.3 (6.1)	23.8 (6.9)	24.8 (7.2)	102.0 (20.9)	104.4 (26.0)
	M	10	182.0 (6.7)	74.9 (15.8)	15.4 (7.2)	37.5 (13.1)	37.4 (11.9)	120.8 (35.4)	131.3 (29.4)
20-29	F	32	167.2 (6.7)	64.4 (15.9)	29.6 (6.8)	23.9 (6.7)	24.9 (7.8)	103.7 (19.4)	106.9 (23.2)
	M	27	177.8 (4.9)	74.9 (10.3)	17.3 (5.7)	39.5 (10.5)	41.9 (9.9)	121.9 (25.7)	131.0 (26.2)
30-39	F	31	164.5 (5.8)	62.8 (10.1)	28.5 (7.9)	24.4 (5.6)	26.6 (6.2)	107.1 (23.2)	113.7 (19.8)
	M	32	176.5 (6.6)	76.4 (12.9)	18.7 (7.2)	34.3 (9.8)	34.5 (8.5)	116.6 (25.7)	123.2 (28.1)
40-49	F	32	163.8 (5.0)	62.4 (8.9)	28.5 (8.3)	25.0 (6.2)	24.9 (6.3)	98.4 (20.9)	102.6 (21.7)
	M	26	176.4 (6.1)	77.3 (12.9)	17.9 (5.5)	39.8 (9.2)	40.6 (9.7)	118.5 (26.3)	132.1 (25.5)
50-59	F	29	162.2 (6.1)	63.9 (10.7)	29.8 (7.6)	23.4 (5.2)	23.5 (5.7)	86.3 (20.2)	97.8 (21.9)
	M	11	178.3 (7.4)	78.8 (10.2)	19.0 (4.6)	39.7 (8.6)	40.5 (7.0)	113.2 (10.5)	116.5 (21.4)
60-69	F	21	160.2 (7.3)	62.8 (10.6)	29.8 (6.8)	21.3 (5.4)	19.2 (4.4)	90.2 (25.6)	96.2 (23.6)
	M	11	172.7 (6.8)	84.7 (13.0)	24.2 (5.5)	31.6 (9.3)	35.1 (11.8)	104.6 (29.5)	112.6 (29.7)
70-80	F	17	161.3 (5.0)	62.8 (8.1)	28.8 (7.8)	21.4 (6.2)	20.9 (7.0)	75.6 (21.0)	83.7 (18.4)
	M	5	173.2 (5.0)	84.3 (13.1)	22.8 (6.2)	39.5 (15.6)	38.2 (13.9)	117.5 (21.9)	112.3 (6.9)

The measurements took approximately 20 minutes to perform. Subjects complied well but many did not develop their maximal voluntary contraction on the first trial and a few felt discomforts during the plantar-flexion measurement due to very large torque of this function and interaction of the strap at the distal part of the thigh. Some subjects (approximately 8%) complained of cramps during the plantar-flexion test and the experiment had to be interrupted for the muscles to rest.

### Normative data

The comparison of torque values between the dominant and non-dominant sides in both right and left-handers is shown in Table 2. Right-handers were significantly stronger for both ankle dorsi-flexion and plantar-flexion measurements on their dominant side, i.e. the right side. Left-handers were significantly stronger for ankle plantar-flexion measurements on their non-dominant side, i.e. the right side. No significant difference between dominant or non-dominant side could be found for dorsi-flexion measurements in left-handers. These differences represent 3 and 7% respectively between right and left dorsi-flexion and plantar-flexion torque values. As the right leg was significantly stronger for both groups, hand dominance does not seem to be a good predictor of the stronger side for both dorsi-flexion and plantar-flexion. Therefore, for the following analyses, the right and left sides were analysed separately without taking into account dominance. Torque values are therefore given for each function and each side (Table 1).

**Table 2:** Differences in torque values between dominant and non-dominant sides

	**Dorsi-flexion**		**Plantar-flexion**	
	**Right handers**	**Left handers**	**Right handers**	**Left handers**
Mean Dominant-Non dominant difference (N⋅m)	0.87	0.63	6.34	-9.01
p-value (paired t test)	<0.01	0.42	<0.01	<0.01
Number of subjects in group	312	33	295	30

### Predictive model

**Figure 2:**
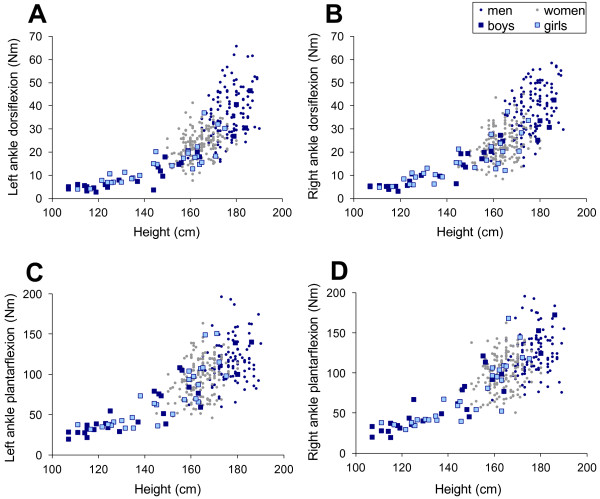
Evolution of torque with height.

Height was the main variable influencing both dorsi-flexion and plantar-flexion strength in both children and adults (Figure 2). Other factors influencing strength for adults were age and gender. Predictive equations better fitted the data after transforming strength values using a natural logarithmic function. The results of modeling are given in Table 3 for children and Table 4 for adults. The R-squared were very good for children models for both dorsi-flexion and plantar-flexion measurements (above 0.80) which indicate a very good fit of the model. They were however lower for the adult models, 0.47 for dorsi-flexion and 0.27 for plantar-flexion measurements, indicating that the model is not able to precisely describe the variability of the data.

**Table 3:** Predictive models for children ankle dorsi- and plantar-flexion torque (N⋅m)

**Logarithmic transformation of muscle function**	**Intercept**	**Height (cm)**	**Adjusted R**^**2**^	**SD**
Left ankle dorsi-flexion	-1.665*	0.0286*	0.826	0.288
Right ankle dorsi-flexion	-1.430*	0.0276*	0.831	0.274
Left ankle plantar-flexion	0.844*	0.0224*	0.805	0.237
Right ankle plantar-flexion	0.716*	0.0237*	0.837	0.225

Models apply to the natural logarithmic transformation of the data.

* p-value<0.05.

**Table 4:** Predictive models for adults ankle dorsi- and plantar-flexion torque (N⋅m)

**Logarithmic transformation of muscle function**	**Intercept**	**Height (cm)**	**Age (years)**	**Sex (0 - 1)#**	**Adjusted R**^**2**^	**SD**
Left ankle dorsi-flexion	0.519	0.01596*	-0.000088	0.2419*	0.464	0.262
Right ankle dorsi-flexion	0.637	0.01573*	-0.001958	0.2659*	0.480	0.271
Left ankle plantar-flexion	3.580*	0.00698*	-0.004260*	0.0983*	0.272	0.226
Right ankle plantar-flexion	3.735*	0.00618*	-0.003320*	0.1121*	0.261	0.214

Models apply to the natural logarithmic transformation of the data.

# 1 for men and 0 for women.

* p-value<0.05.

### Reliability assessment

**Figure 3:**
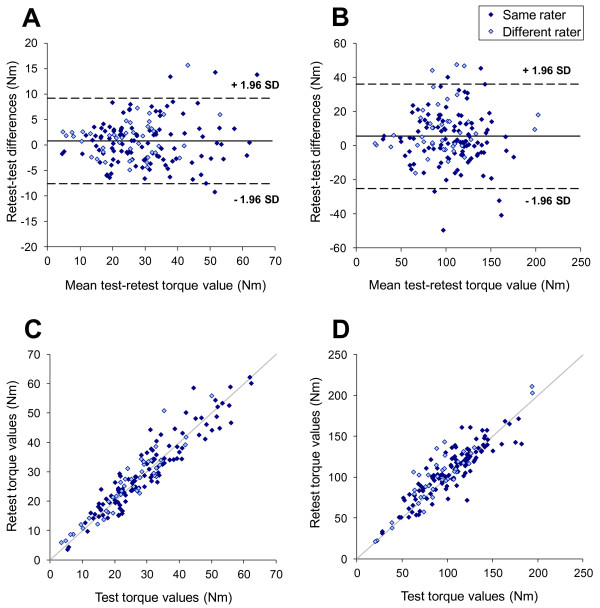
Bland & Altman and correlation plots for ankle dorsiflexion (A and C) and plantarflexion (B and D).

Seventy six healthy subjects performed a second ankle strength session. All had dorsi-flexion measurements and 74 had plantar-flexion measurements. Fifty four subjects were evaluated by the same evaluator during the retest session whereas 22 subjects were evaluated by a different evaluator. For both dorsi-flexion and plantar-flexion, no evaluator effect was found. For all subjects having retest sessions, there was a significant “visit” effect and a “side” effect but no interaction between both. The analysis of the “visit” effect was therefore performed for each function, taking into account, neither the “evaluator” effect nor the “side” effect. There were 0.8 N⋅m and 5.5 N⋅m significant differences between retest and test dorsi-flexion measurements and plantar-flexion measurements, respectively (refer to Table 5). These differences represent an increase of 3 and 5% for ankle dorsi-flexion and plantar-flexion strengths, respectively, between test and retest sessions. The standard error of measurement (SEM) was 3.0 N⋅m for dorsi-flexion measurements and 11.0 N⋅m for plantar-flexion measurements. These errors represented a coefficient of variation of about 11% for both measurements. The limits of agreement between two successive measurements were 8.4 N⋅m for dorsi-flexion measurements and 30.6 N⋅m for plantar-flexion measurements. These values represent the smallest detectable difference (SDD), i.e. the smallest difference that would indicate a real difference between the measurements with a 95% confidence [30] for healthy subjects. The results are shown as a Bland and Altman graph and a correlation graph on Figure 3 (A and B). The retest correlation analysis showed a good correlation for both ankle dorsi-flexion and plantar-flexion measurements as shown in Figure 3 (C and D). ICCs were 0.94 for dorsi-flexion and 0.88 for plantar-flexion.

**Table 5:** Test-retest agreement and reliability

**Parameters**	**Ankle dorsi-flexion**	**Ankle plantar-flexion**
Number of subjects	76	74
Mean difference (N⋅m)	0.76	5.46
p-value (paired t-test)	0.03	<0.0001
SEM (N⋅m)	3.0	11.0
Relative SEM (%)	11	11
Limits of agreement (N⋅m)	8.4	30.6
Intra Class Correlation Coefficient	0.94	0.88

SEM= Standard Error of Measurement.

### Feasibility in a small patient group

**Figure 4:**
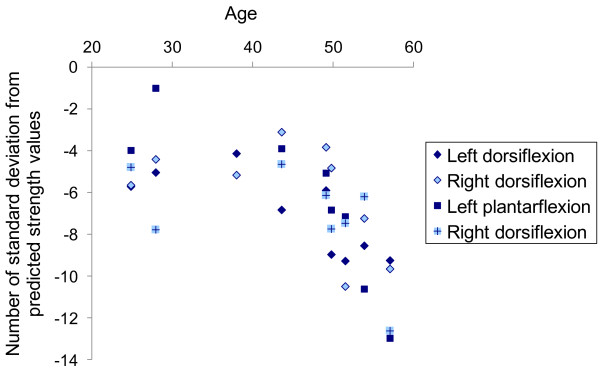
Evolution of the defect of strength (z-scores) with age in patients with LGMD2A.

Nine patients with calpainopathy performed ankle strength measurements. One patient was not able to perform ankle plantar-flexion measurements. Predictive values were computed according to models described in Table 4. Strength, as expressed as z-scores, decreased with age in older subjects who had a lower strength than younger subjects (Figure 4). There seems to be a cut-off around 45 years of age where strength decreases rapidly. Values as low as 1.1 N⋅m could be recorded showing that the device can detect strength in very weak patients. The mean (SD) z-scores were -7.1 (2.0) and -6.0 (2.6) for left and right ankle dorsi-flexion respectively. For ankle plantar-flexion, the mean (SD) z-scores were -6.4 (3.9) and -7.2 (2.5) for the left and right sides respectively.

The Vignos lower extremity scale was significantly negatively correlated with the mean strength z-score (Pearson R=-0.851; p=0.002).

## Discussion

Ankle strength is an important factor for ambulation and balance [1,2] and should therefore be closely monitored. A novel dynamometer has been developed in order to accurately measure both ankle dorsi-flexion and plantar-flexion strength in children and adults with neuromuscular disorders. Normative data were established and predictive models computed for both children and adults. The device was shown to have a good level of reliability with low relative standard errors of measurement between test and retest sessions. The feasibility to use this novel dynamometer on patients was confirmed on a small sample of patients affected with calpainopathy.

### Feasibility of the measurements

The device was both easy to use and transport. Its size makes it usable for children, as young as five years old, as well as for elderly people as old as eighty years of age. The device allows one to measure torque values up to 200 N⋅m with a very good stabilization of the joint even for ankle plantar-flexion which is a function difficult to measure due to the extent of its strength, even for children [12]. A few subjects complained of discomforts but none refused to perform the measurements. The reluctance of some to perform at their maximal strength on the first trial may be partly explained by the apprehension of the subjects to hurt themselves.

### Normative data

We found that the right side was significantly stronger than the left side independently of the laterality determined by hand dominance. The hand dominance does not seem to be a good indicator for leg dominance. Indeed the recommendation given in the literature is to establish foot preference by asking the subject which leg he or she would choose to kick a ball [31]. This method was applied by several authors who found that there was no significant differences in force between the dominant and the non-dominant side in dorsi-flexion strength [20,32] or between the left and right leg [20]. However we found that the right side was significantly stronger than the left side; this result is comparable to that obtained by other authors [25,33].

In adults, the dorsi-flexion torque values found in this study are comparable to those found in other studies where the ankle joint was flexed at 90° [18,20,34]. In other studies, where the ankle joint was not chosen at 90°, dorsi-flexion and plantar-flexion torque values may differ slightly [13,14,27]. These differences observed between different studies may be attributed to differences in the population tested and to the test protocol itself especially the positioning of the subject. Indeed, ankle plantar-flexion torque has been demonstrated to be greater when the knee was extended rather than flexed to 90° [21-23]. Moreover it was shown that ankle plantar-flexion torque is maximal when the ankle joint is dorsiflexed by 15° [23] and ankle dorsi-flexion torque is maximal when the ankle joint is plantarflexed by 10-15° [16,19]. In children, results for dorsi-flexion and plantar-flexion torque values in our study differ slightly from other studies [12,24] probably due to discrepancies in test protocols already mentioned above and due to difference in stature that can have a great impact on strength.

Torque values found in our study are therefore probably lower than the maximum value possible when both the ankle and knee position are optimized. However, the seated position was chosen over other positions in order to minimize the possibilities of co-contractions and to enable as many subjects as possible to perform the measurements even in the presence of contractures.

### Predictive models

Most authors have used age, weight, sex and sometimes height as predictive variables for ankle strength [12,32,34,35]. In this study we found that height was the best predictor of strength for both children and adults and that other variables added little to the model. Using height to predict strength in children may enable one to assess muscle weakness in children with muscle disabilities associated with growth disorders. If a child is small for his age, prediction will make it possible to establish the part of muscle weakness that is due to disease and not growth retardation. Eek et al. [12] considered age, weight and sex as the predicting variables of ankle dorsiflexors strength in children and found determination coefficients comparable to ours. They indicated that sex was included in the model when the age was over 13. As Hogrel et al. [36], we found that when height was taken into account, no gender effect was seen in the strength of children. In adults, the model predicting dorsi-flexion torque in our study has similar determination coefficients compared to other models [32,34,35]. The quality of our plantar flexion model could not be assessed either in children or adults as no other study computing this model could be found. The difference in determination coefficients between dorsi-flexion and plantar-flexion models found in our study can be interpreted as a higher variability in plantar-flexion strength rather than dorsi-flexion strength among adults. Even though our model only explains a small amount of plantar-flexion variability, it may be useful in evaluating strength deficit for this function as, to our knowledge, no other model has been established.

### Reliability assessment

The significant positive differences between retest and test sessions suggest that there might be a minor learning effect in performing maximal voluntary contractions for ankle movements. This phenomenon may also be explained by the apprehension of the subject to hurt himself during the first trial as was previously mentioned or by the fact that this function is not performed isometrically in everyday life and can therefore be difficult to perform at first. This increase in strength during the retest session on plantar-flexion strength measurements has been observed by several authors [14,15]. Standard error of measurements and ICCs found in our study are similar or higher to those found in other studies assessing reliability in dorsi-flexion or plantar-flexion strength [14,25,34]. Due to the possible learning effect in assessing ankle strength, a training session should be recommended in clinical trials where there is a follow up of patient strength over time. A training session would also be useful in evaluating the smallest detectable differences that is relevant to the patient population studied. Indeed the smallest detectable difference found in our study was established in healthy subjects and may not be a good criterion for detecting changes in subjects with much lower strength values as it can be noticed in our results (see Figure 3) that the difference between test and retest session is higher for higher strength values. Moreover, the variations in subsequent measurements depend on motivation, skill, learning effect and several individual variables that are complex to analyse. This can affect estimates of reliability.

### Feasibility in a small patient group

The dynamometer was applied without particular difficulties to nine patients with limb girdle muscular dystrophy. Values of maximal torque as low as 1.0 N⋅m could be recorded. Using the predictive equations established in this study, all subjects were shown to have a significant strength deficit compared to the normal population. Therefore these quantitative strength measurements relative to the normal population highlight the strength deficit of these patients in a quantitative way. The device could therefore be used in clinical trials for the follow up of different pathologies in which leg muscles are affected, even severely. Note that most of the patients present a strength that lies under the smallest detectable difference, which may be of limited pertinence in this case.

### Study limitations

Not all subjects had a retest session, meaning that norms and predictive equations were established on test values which might not be representative of the maximum values as a learning effect was demonstrated on a subgroup of patients.

## Conclusions

The device we have developed shows a good level of reliability and accuracy in assessing ankle dorsi-flexion and plantar-flexion torque measurements from weak patients to strong healthy adults. The size of the device makes it useable for both children and adults. This device overcomes the drawbacks of other measuring methods such as sensitivity to low values of strength and joint stabilization.

## Abbreviations

MMT: Manual muscle testing; LGDM2A: Limb girdle muscular dystrophy type 2A; MVC: Maximal voluntary contractions; CVar: Coefficient of variation; ICC: Intraclass correlation coefficient; SDD: Smallest detectable difference; SEM: Standard error of measurement.

## Competing interests

The authors have no competing interest.

## Authors’ contributions

AM analysed the data and drafted the manuscript. AC, GO and IL performed the experiments and drafted the manuscript. CP and VD organized the experiments and drafted the manuscript. JYH was the principal investigator of the study and designed the experiments, performed the experiments, analysed the data and drafted the manuscript. All authors read and approved the final version of the manuscript.

## Pre-publication history

The pre-publication history for this paper can be accessed here:

http://www.biomedcentral.com/1471-2474/14/104/prepub
